# Androgen receptor and its splice variant, AR-V7, differentially induce mRNA splicing in prostate cancer cells

**DOI:** 10.1038/s41598-021-81164-0

**Published:** 2021-01-14

**Authors:** Manjul Rana, Jianrong Dong, Matthew J. Robertson, Paul Basil, Cristian Coarfa, Nancy L. Weigel

**Affiliations:** 1 Department of Molecular and Cellular Biology, Baylor College of Medicine, Houston, TX 77030 USA; 2 Dan L. Duncan Cancer Center, Baylor College of Medicine, Houston, TX 77030 USA; 3 Present Address: Mercury Data Science, Houston, TX 77098 USA

**Keywords:** Endocrine cancer, Transcriptomics

## Abstract

Prostate cancer (PCa) is dependent on the androgen receptor (AR). Advanced PCa is treated with an androgen deprivation therapy-based regimen; tumors develop resistance, although they typically remain AR-dependent. Expression of constitutively active AR variants lacking the ligand-binding domain including the variant AR-V7 contributes to this resistance. AR and AR-V7, as transcription factors, regulate many of the same genes, but also have unique activities. In this study, the capacity of the two AR isoforms to regulate splicing was examined. RNA-seq data from models that endogenously express AR and express AR-V7 in response to doxycycline were used. Both AR isoforms induced multiple changes in splicing and many changes were isoform-specific. Analyses of two endogenous genes, PGAP2 and TPD52, were performed to examine differential splicing. A novel exon that appears to be a novel transcription start site was preferentially induced by AR-V7 in PGAP2 although it is induced to a lesser extent by AR. The previously described AR induced promoter 2 usage that results in a novel protein derived from TPD52 (PrLZ) was not induced by AR-V7. AR, but not AR-V7, bound to a site proximal to promoter 2, and induction was found to depend on FOXA1.

## Introduction

The androgen receptor (AR, NR3C4) is a hormone activated transcription factor that is important for the growth, invasion, and progression of prostate cancer (PCa)^1–3^. Metastatic PCa is treated with an androgen deprivation therapy (ADT) based regimen but eventually, these tumors develop resistance through multiple mechanisms that reactivate AR^4–6^. These include the expression of constitutively active AR splice variants that lack a ligand-binding domain such as AR-V7^6–10^. Whether and how AR-V7 differentially regulates transcription relative to AR has been a subject of great interest, but its capacity to regulate splicing has not been studied.

Alternative splicing is broadly recognized for its vital role in the regulation of genes and creating gene diversity in multicellular organisms^11,12^. Over 90% of human genes undergo alternative splicing^13,14^. Different alternative combinations of exons and splice sites generated during splicing can produce multiple transcripts and different protein sequences from a single locus. This is, in part, responsible for the much greater diversity of the transcriptome and proteome than would be obtained from a single protein isoform derived from each gene^13,15,16^. Dysregulation or defects in the process may lead to disruption of normal cellular function and the eventuality of disease including cancer^17–19^.

There are many types of alternative splicing; the most common are exon skipping (ES), mutually exclusive exons (MXE), alternative 5′ splice sites (A5SS), alternative 3′ splice sites (A3SS), alternative first exon (AFE), alternative last exon (ALE), tandem 3′UTRs and intron retention (IR)^20,21^. Exon skipping events are reported to be the most prevalent alternative splicing event in the human genome and aberrant splicing associated with it is responsible for the loss of functional domains or a frameshift in the coding sequence^22,23^.

Besides controlling transcriptional levels, steroid hormone receptors play a role in regulating alternative splicing^4,24–27^. RNA-seq based data of AR activity in response to androgens identified many genes whose transcription is regulated by the AR in prostate cancer^26^. Transcription also has been coupled with pre-mRNA processing and promoter selection in the presence of androgens in prostate cancer^26,28–31^. Previously, we prepared AR expressing LNCaP and VCaP prostate cancer cell lines that express AR-V7 in response to Doxycycline (Dox)^32,33^. Analysis of our RNA-seq data and other studies revealed differential expression of target genes (GEO#GSE143905^33^). Our current analyses reveal that AR activated by R1881 or AR-V7 induced by Dox drive the production of alternative mRNA isoforms in prostate cancer cell lines. This includes all forms of alternative splicing resulting in changes in both coding and regulatory regions in untranslated regions. Here, we have identified differential alternative splicing events induced by the AR isoforms in LNCaP and VCaP cell line models and evaluated the mechanisms for differential induction.

## Results

### Expression of the AR-V7 variant in the inducible LNCaP and VCaP prostate cancer cell line models

**Figure 1:**
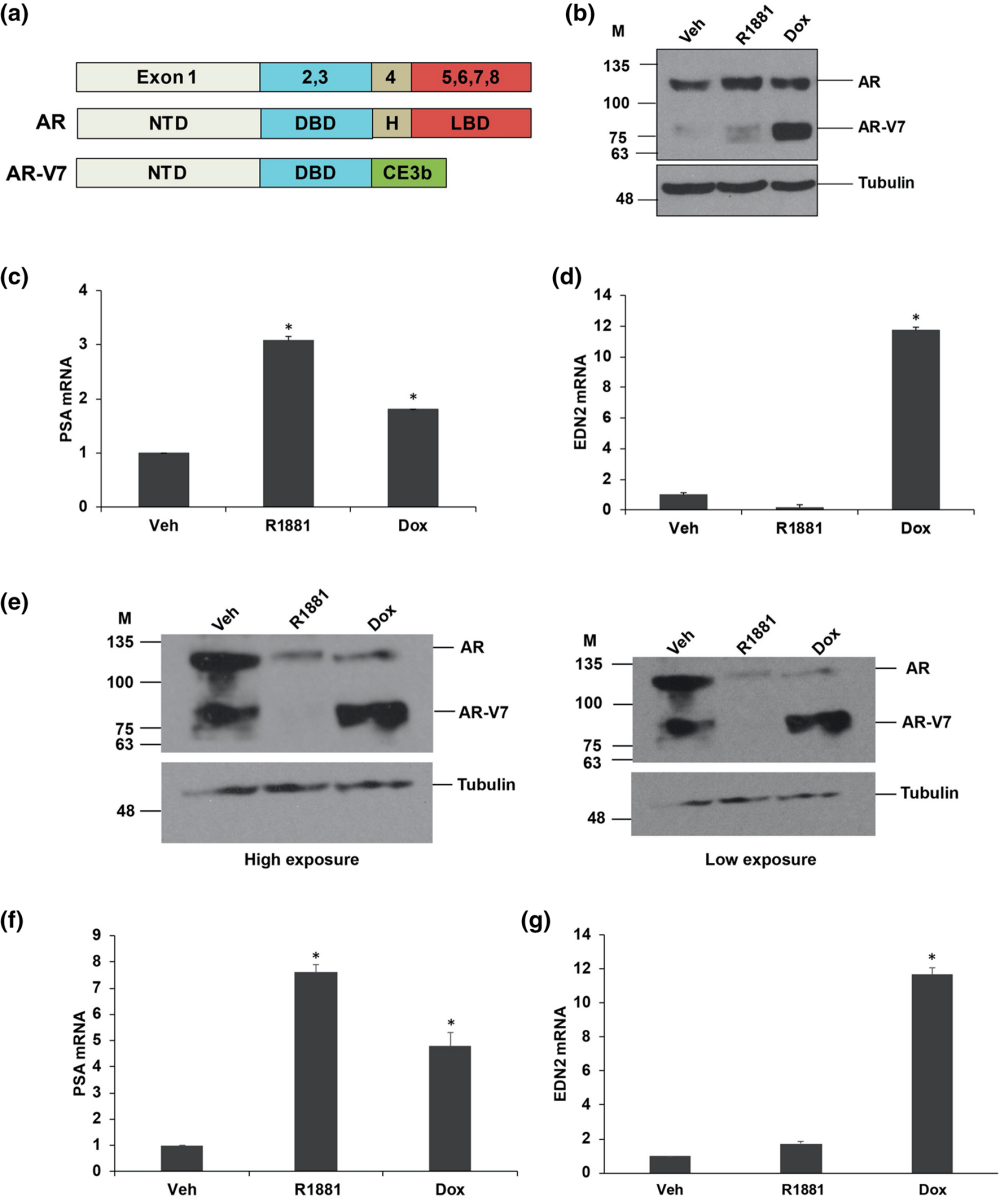
Expression of the AR-V7 variant in LNCaP-AR-V7 and VCaP-AR-V7 cells. (**a**) Schematic representation of full-length AR and AR-V7 variant showing the exon structure and protein domain structure NTD, N terminal domain; DBD, DNA binding domain; H, hinge region; LBD, ligand-binding domain. (**b**) LNCaP-AR-V7 cells were changed to medium containing charcoal-stripped serum (CSS) and treated with vehicle (EtOH), R1881 (10 nM), or Doxycycline (Dox) (20 ng/ml for LNCaP-AR-V7) for 24 h and cells harvested for western blot analysis with AR441 and tubulin antibodies. (**c**, **d**) LNCaP-AR-V7 cells were treated as described in (**b**), RNA isolated and AR target gene PSA, and the AR-V7 specific target gene, EDN2 were measured using RT-qPCR. (**e**) VCaP-AR-V7 cells were changed to medium containing charcoal-stripped serum (CSS) and treated with vehicle (EtOH), R1881 (10 nM), or Doxycycline (Dox) (100 ng/ml for VCaP-AR-V7) for 24 h and cells harvested for western blot analysis with AR441 and tubulin antibodies (high and low exposures are shown). (**f**, **g**) VCaP-AR-V7 cells were treated as described in (**e**), RNA isolated and AR target gene PSA, and the AR-V7 specific target gene, EDN2 were measured using RT-qPCR. Expression values were plotted as a mean of three biological replicates from the same experiment. Error bars represent SEM. Asterisks (*) signify values that differed significantly with respect to vehicle (*P* < 0.05 in Student's t-test).

The structure of full-length androgen receptor (AR) and its variant AR-V7 are shown in Fig. 1a. The full-length AR contains 8 exons that encode four functional domains: the domains starting from the amino-terminus are the amino-terminal transactivation domain (NTD) encoded by exon 1, a DNA binding domain (DBD) which is encoded by exon 2 and exon 3, a flexible domain called the hinge region encoded by the 5′ portion of exon 4 and a C-terminal domain called ligand-binding domain (LBD) encoded by exons 4–8^34^. AR-V7 lacks the LBD and hinge region, but contains the amino-terminal transactivation domain and DNA binding domain (DBD) encoded by exons 1–3 and 16 unique amino acids derived from a cryptic exon 3b^8,9^. We have previously described the LNCaP and VCaP models in which AR-V7 can be induced using Doxycycline^32,33^. The levels of expression of full-length AR and induced AR-V7 protein are shown in Fig. 1b in LNCaP-AR-V7 and in Fig. 1e in VCaP-AR-V7 cells. Note that VCaP cells do express some endogenous AR-V7 (Fig. 1e), but Dox treatment substantially increases expression. Activation of AR by R1881 in VCaP-AR-V7 cells causes downregulation of both endogenous AR isoforms as previously reported^35,36^; whereas Dox induced expression of AR-V7 also represses expression of endogenous AR (Fig. 1e). The characteristic induction of PSA by AR and AR-V7 is shown in Fig. 1c,f and the preferential induction of EDN2^33^ by AR-V7 is shown in Fig. 1d,g in LNCaP-AR-V7 and VCaP-AR-V7 respectively.

### Analysis of the alternative splicing events in the LNCaP and VCaP models

**Figure 2:**
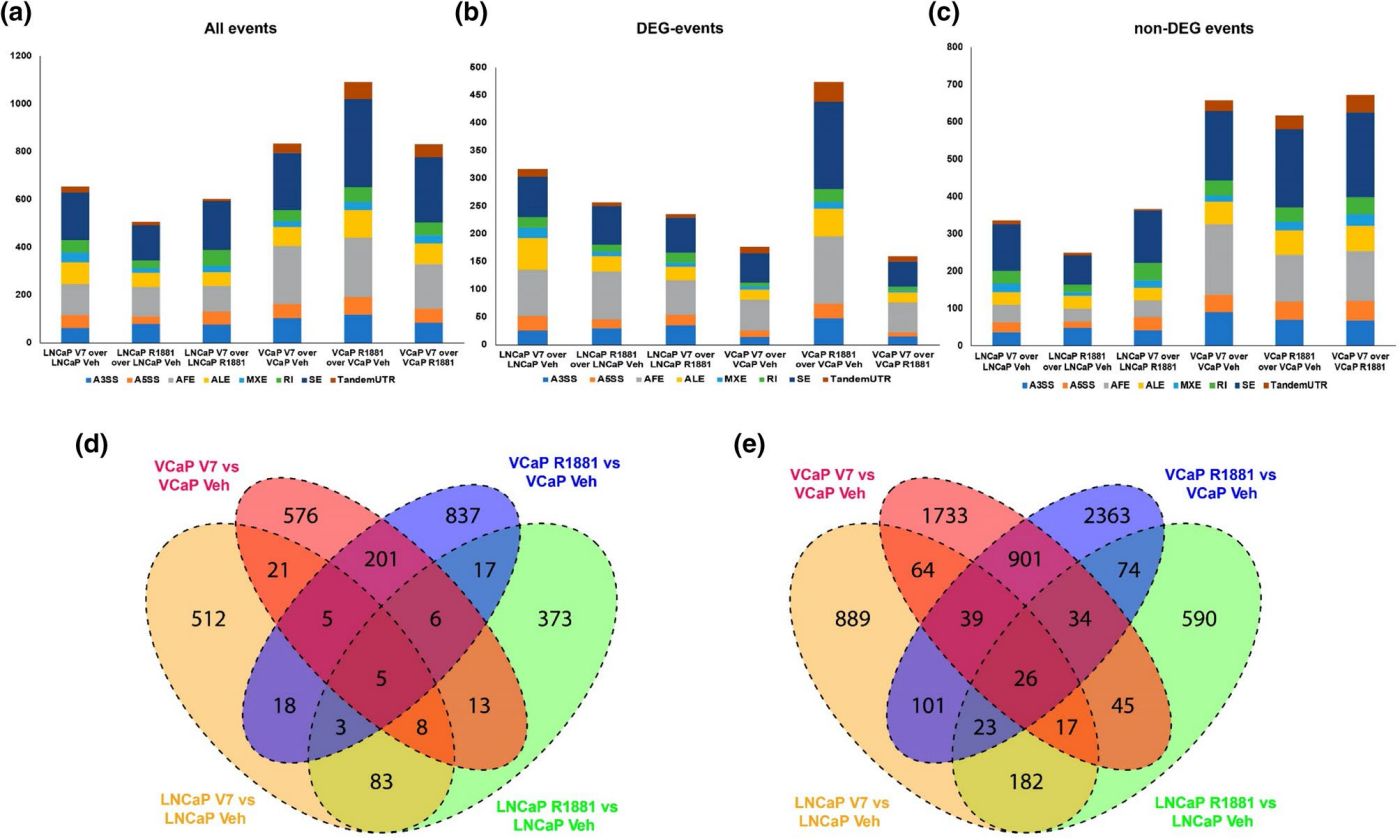
Analysis of the alternative splicing events in the LNCaP and VCaP models. The number of each type of event derived from the reads of RNA-seq data as described in methods of LNCaP treated with R1881 (AR full length), VCaP treated with R1881, LNCaP- AR-V7 treated with Doxycycline and VCaP-AR-V7 treated with Doxycycline relative to the corresponding vehicle. (**a**) All events (total); (**b**) events in regulated genes (DEG), and (**c**) events in non-regulated genes (non-DEG). A3SS, alternative 3′ splice site; A5SS, alternative 5′ splice site; AFE, alternative first exons; ALE alternative last exons; MXE, mutually exclusive exons; RI, retained introns; SE, skipped exons; Tandem UTR, Tandem 3′UTRs. (**d**) Overlap of splicing events in the two cell lines and for each AR isoform studied with Bayesian Factor (BF) ≥ 3 and changes in fraction isoform abundance (ψ) between treatment conditions greater than 0.2 (Δψ > 0.2). (**e**) Overlap of splicing events in the two cell lines and for each AR isoform studied with Bayesian Factor (BF) > 3 and no cut-off for change in fraction isoform abundance (ψ) between treatment conditions (Δψ = 0).

Alternative splicing events were identified in the entire read set for the RNA-seq data from our LNCaP-AR-V7 and VCaP-AR-V7 models (STable 3). Splicing events were identified and quantified as described in methods. Events with a Bayesian Factor (BF) ≥ 3 and changes in fraction isoform abundance (ψ) between treatment conditions greater than 0.2 (Δψ > 0.2) were analysed in panels Fig. 2a–d. Figure 2a shows the number and types of events induced by AR and AR-V7 in both models regardless of whether they are in differentially expressed genes (DEG) or non-regulated genes (non-DEG). Figure 2b,c show the subset of events in DEG and non-DEG respectively. Note that there is substantial differential splicing in genes whose net expression is not considered to be regulated by the AR isoforms as well as in regulated genes. The most common event type is the exon skipping (SE) event (Fig. 2a). For the LNCaP lineage, the number of events in DEG and non-DEG genes is similar whereas in the VCaP models there are greater number of events in vehicle or DOX treated non-DEG compared to DEG (Fig. 2b,c). For the LNCaP model there is around two-fold higher proportion of Alternative first exon (AFE) events in DEG compared to non-DEG. This has not been observed in the VCaP model. Figure 2d shows the overlap in events between the two cell lines. Only a small fraction of LNCaP-AR-V7 events (~ 6%) are also regulated by AR-V7 in VCaP cells. When the alternative splicing data were evaluated for events with a Bayesian Factor (BF) > 3 and with no restriction on fraction isoform abundance (ψ) between treatment conditions (Fig. 2e), the total numbers of splicing events increased and there was a small increase in relative overlap between LNCaP AR-V7 and VCaP AR-V7 (~ 11% of events).

### Skipped Exon event in PGAP2

**Figure 3:**
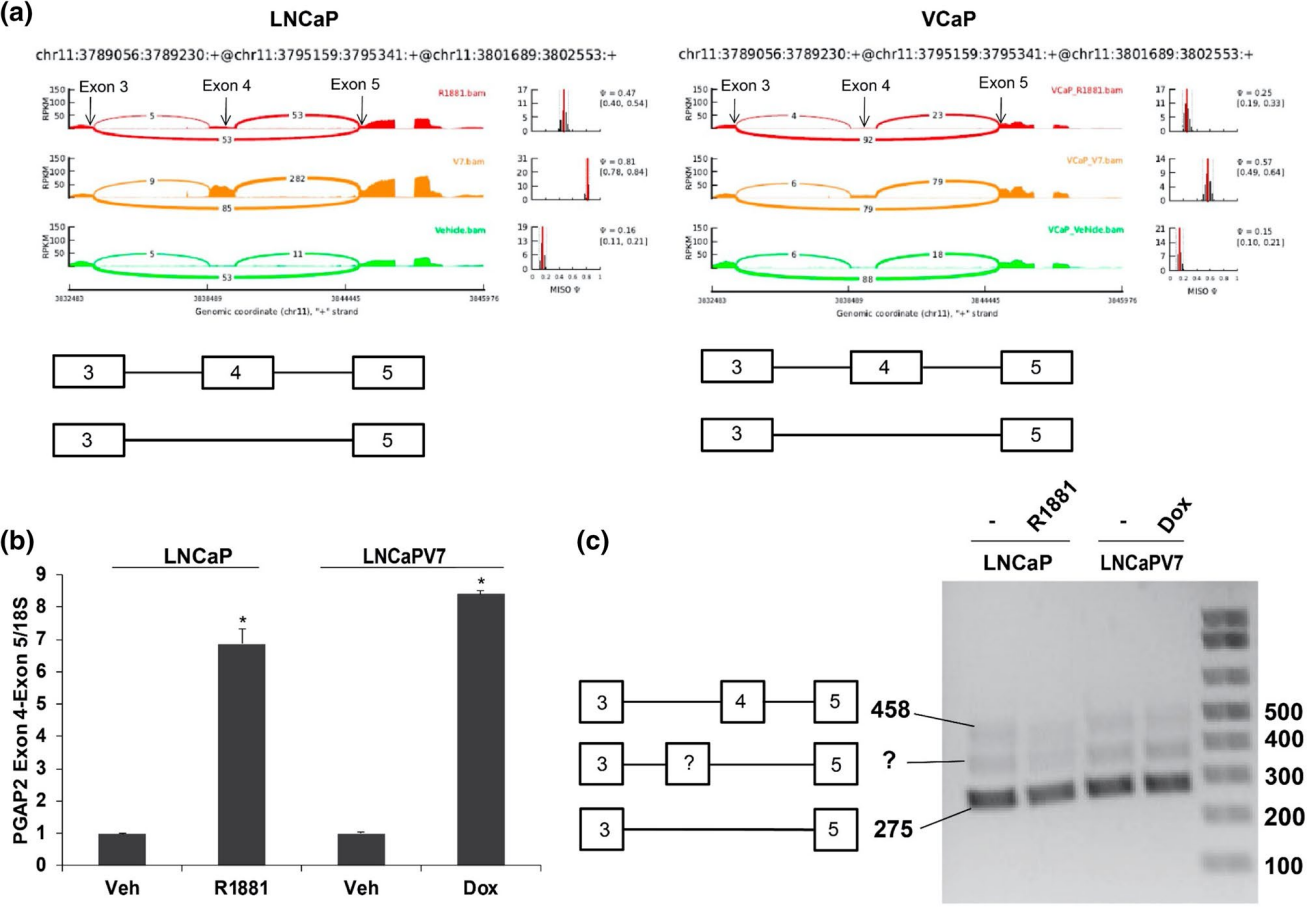
Skipped exon event in PGAP2. (**a**) Sashimi plots for PGAP2 inclusion/exclusion of exon 4 in the LNCaP and VCaP models. To analyze the expression of the included and excluded forms, LNCaP and LNCaP-AR-V7 cells were transferred to CSS medium and treated with vehicle, R1881, or Dox for 24 h, and RNA was isolated. (**b**) qPCR analysis of the region encompassing Exon 4 and 5 (long isoform) in the LNCaP model showed the expected increase in expression. Expression values were plotted as a mean of three biological replicates from the same experiment. Error bars represent SEM. Asterisks (*) signify values that differed significantly with respect to vehicle (*P* < 0.05 in Student's t-test). (**c**) Agarose gel analyses of products amplified using one primer in exon 3 and one in exon 5. The bottom band of 275 bp represents the excluded form; the top band 458 bp represents long (Exon 4 included) form. There is an additional intermediate band suggesting that there is an additional sequence, which may represent an unknown exon*.*

Skipped Exon events represent the most common alternative splicing event type involved in regulating genes^22^. One of the most differentially regulated events in both cell lines (LNCaP-AR-V7 and VCaP-AR-V7) is an inclusion/exclusion exon event in PGAP2 (Post-GPI Attachment to Proteins 2), a protein involved in GPI remodeling. Figure 3a shows the Sashimi plot for PGAP2 for known exons in the exon 3–5 region in both models showing inclusion or skipping of exon 4 in PGAP2. The Sashimi plots suggested that AR-V7 preferentially induced a form that includes exon 4. Analysis of the expression of an exon 4-exon 5 junction by qPCR showed that both isoforms increased inclusion, with AR-V7 inducing slightly higher expression than AR (Fig. 3b). Agarose gel-based analysis showed good expression of the skipped form (Fig. 3c), but very low levels of the included Exon 3, 4 and 5 form and no evidence of induction. A novel intermediate band was also found to be present but was low in abundance.

### A Novel initiation site of transcription exists between Exons 3 and 4 of PGAP2

**Figure 4:**
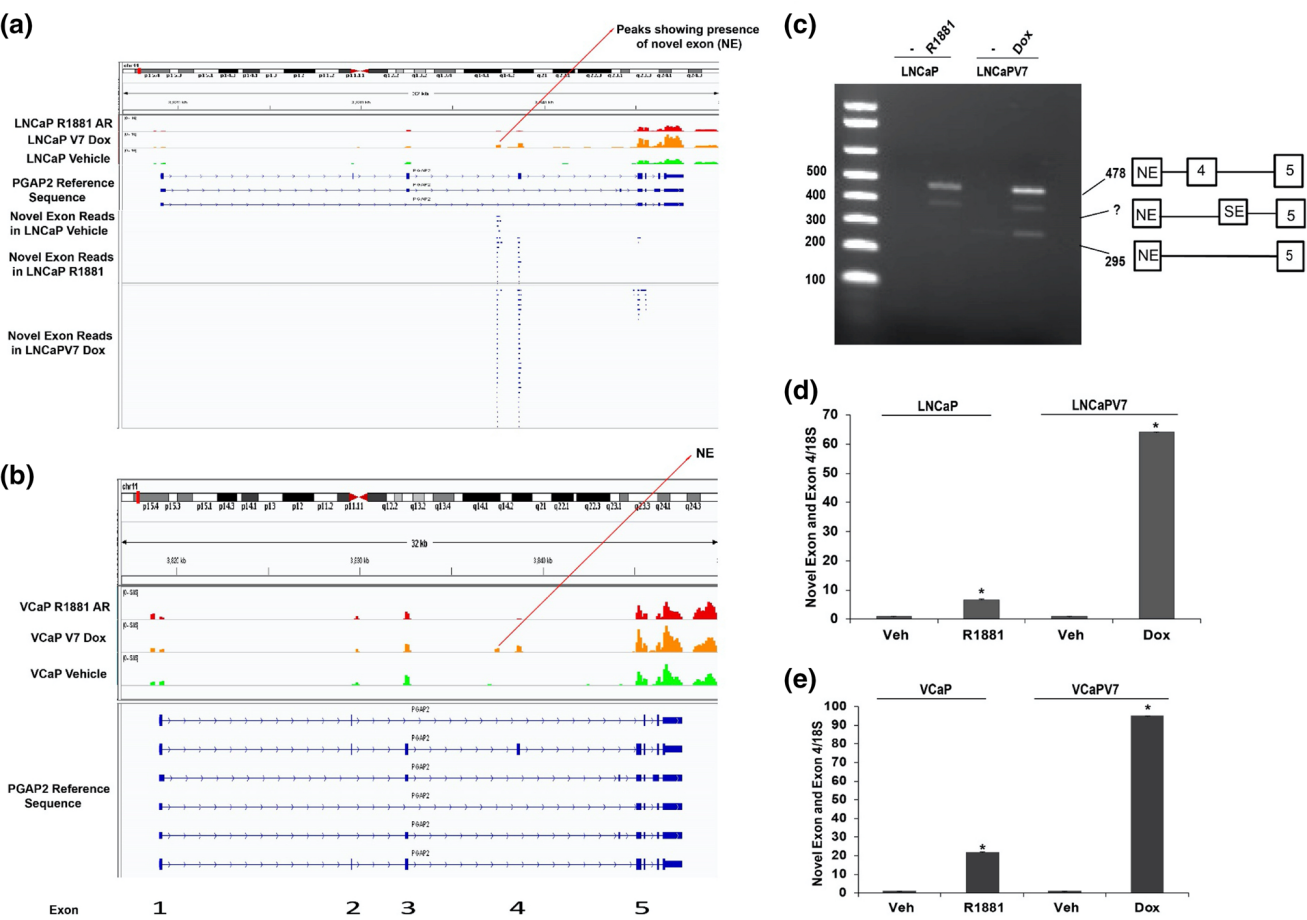
A novel exon exists between exons 3 and 4. (**a**) IGV plot showing the presence of Novel Exon (NE) reads in PGAP2 in the LNCaP cell line and locations of sequence overlap containing a portion of the novel exon. (**b**) IGV peak reads of PGAP2 in the VCaP model also shows a transcribed region between exons 3 and 4. (**c**) Semi-quantitative PCR spanning Novel Exon (NE) and exon 5. The 295 bp band is the excluded form and the top band (478 bp) is the long (Exon 4 included) form. The middle band may correspond to the novel exon linked to another skipped exon between exons 4 and 5 linked to exon 5. (**d**, **e**) qPCR validation of novel exon linked to Exon 4 in LNCaP and VCaP models respectively.

Reviewing the Sashimi plots (Fig. 3a) revealed that the number of reads for the exon 3–4 junction was not nearly as high as the exon 4–5 reads. This led us to examine closely the mapped reads using the Integrative Genome Viewer (IGV) platform. Since the RNA-seq experiment was performed using paired reads, we plotted the ends of each read pair. Figure 4a showed that there is a read pileup corresponding to the region of expression (Novel Exon (NE)) between exons 3 and 4 in the LNCaP model, which does not correspond to a known PGAP2 exon. Read pileups outside the NE correspond to paired reads indicating that the NE is linked to exon 4 and exon 5. This Novel Exon (NE) is also expressed in the VCaP model (Fig. 4b). Agarose gel-based analysis of amplification of the region between the Novel Exon (NE) and exon 5 showed that both R1881 and V7 induced expression of a form that includes exon 4 (Fig. 4c). We observed a band showing direct linkage of the Novel Exon (NE) to exon 5 in LNCaP-AR-V7 samples, and finally found an intermediate form of unknown origin. Figure 4d,e showed quantitative measurements of the induction of a form containing the Novel Exon (NE) and exon 4 in LNCaP and VCaP models respectively. In this case, both isoforms induce this form, but this novel isoform-specific event is induced much more by AR-V7. This raised the question of what the novel exon is linked to on the 5′ end. The gel-based analysis in Fig. 3c showed that there are essentially no forms that contain an insert between exons 3 and 5. An analysis of a potential junction between upstream exons (exons 1, exon 2 and exon 3) and the novel exon did not show any connection relative to the junction with exon 4 (data not shown). Moreover, there is no increase in reads upstream of the novel exon in R1881 or AR-V7 samples (Fig. 4a). This suggests that there is a potential novel initiation site of transcription induced by both R1881 and AR-V7. The length of the novel exon is 75 nucleotides and it would be in frame with the upstream and downstream exons if it were included in the mRNA. However, our data support a novel site of initiation of transcription. In this frame, there are no codons that would provide an initiating methionine, so the likely protein product would begin with the sixth amino acid, a Met, in exon 4 (Supplementary Fig. 1a). Unfortunately, we were unable to confirm this because none of the commercial antibodies tested were sufficiently specific to reliably detect the full-length form.

### Both AR isoforms bind to the novel exon

**Figure 5:**
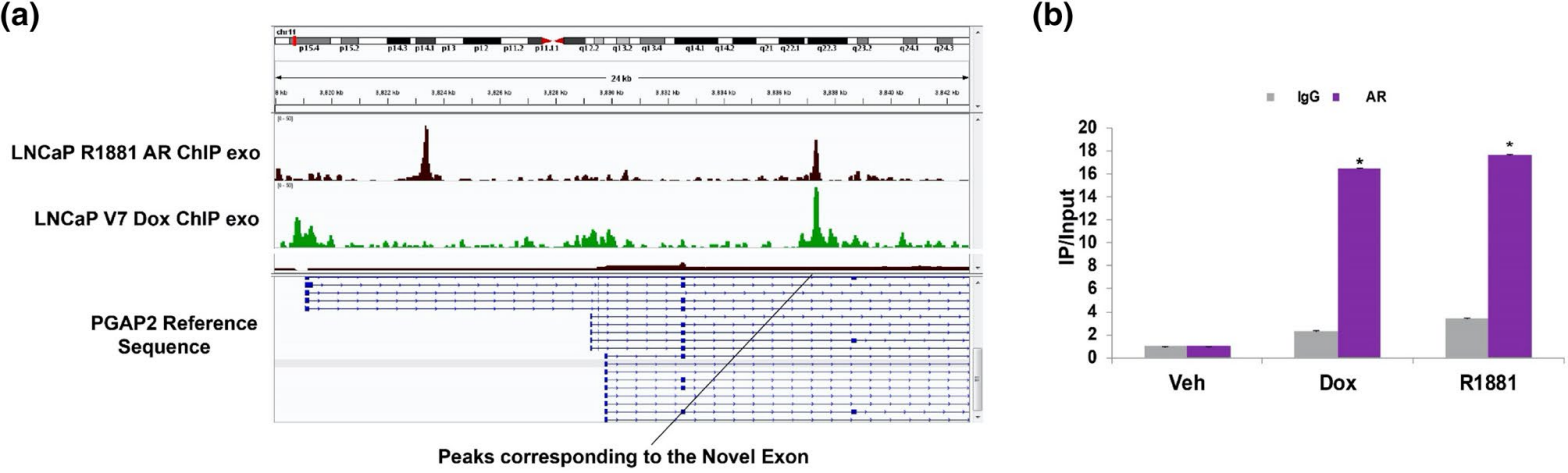
Both AR isoforms bind to the region containing the novel exon. (**a**) shows the ChIP-exo tracks showing binding of AR and AR-V7 to the novel exon region and (**b**) shows ChIP-qPCR validation of AR and AR-V7 binding in LNCaP AR-V7 cells. LNCaP AR-V7 cells were treated with Vehicle, R1881, or Dox and after 24 h ChIP was performed with an amino-terminal AR antibody, and rabbit IgG was used as a negative IP control. Quantitative PCR was performed with primers specific for the novel exon of the PGAP2 gene. It shows the recovery expressed as the relative amount of immunoprecipitated DNA normalized to input DNA after qPCR analysis. Expression values were plotted as a mean of two biological replicates from the same experiment. Error bars represent SEM. Asterisks (*) signify values that differed significantly with respect to immunoprecipitated vehicle (*P* < 0.05 in Student's t-test). Each experiment was performed a minimum of three times and a representative experiment was shown.

Overlap with ChIP-exo data of LNCaP cells expressing either the full-length AR or the AR-V7 isoform showed clear peaks at the location of the novel exon, further suggesting a potential novel transcription initiating site under the control of AR or AR-V7 (Fig. 5a). This was also confirmed by ChIP-qPCR (Fig. 5b) as both isoforms showed strong binding with AR antibody with respect to IgG control.

### Expression of the Novel Exon in LN-95 and 22Rv1 cells

**Figure 6:**
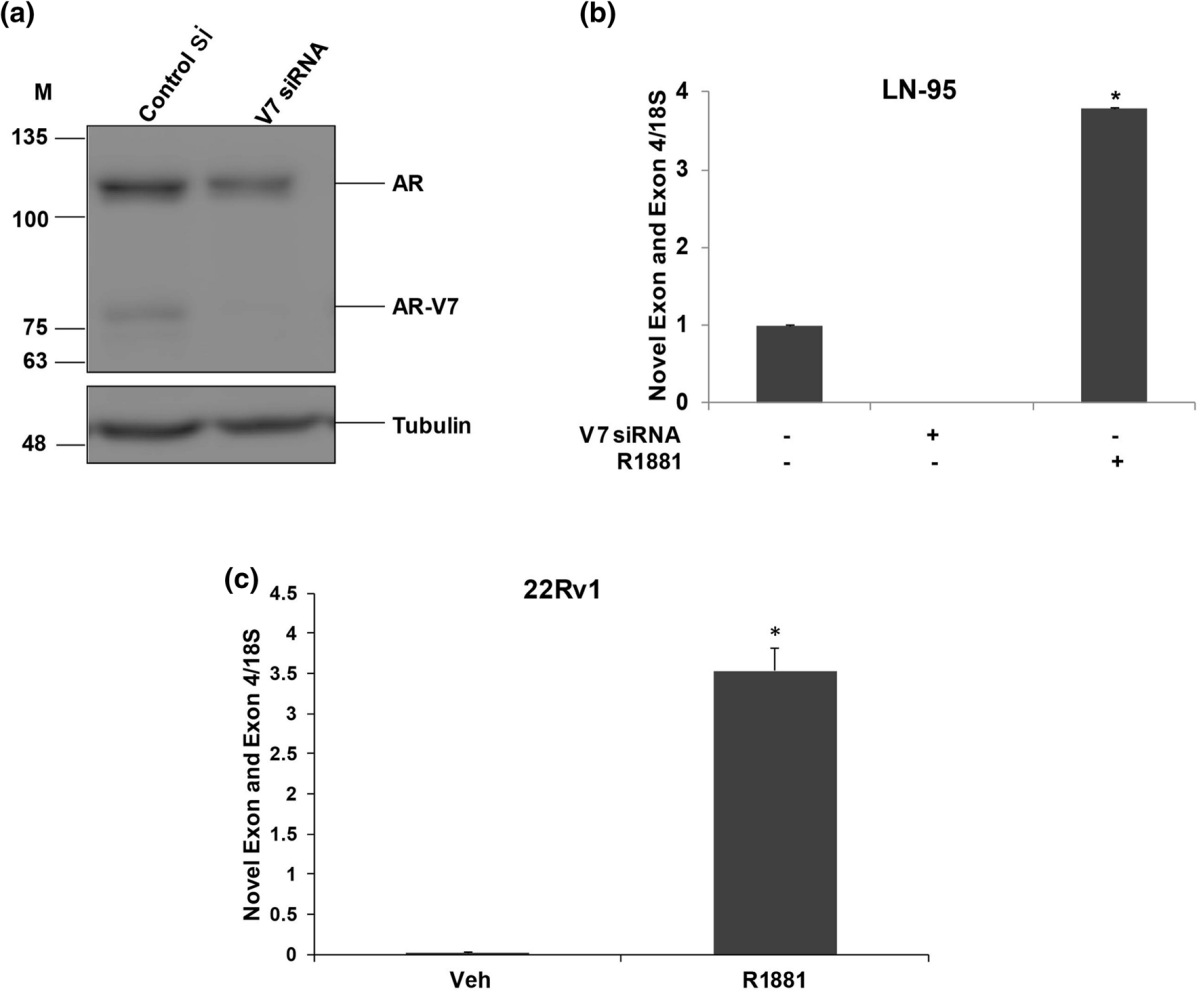
Expression of the Novel Exon in LN-95 and 22Rv1 cells. (**a**) LN-95 cells were treated with control si or AR-V7-siRNA and cells were harvested at 72 h for western blotting and probed with AR 441 and tubulin antibodies. (**b**) LN-95 cells were treated with control si, si-V7, and control si + R1881, and cells were harvested at 72 h and subjected to qPCR with the primers comprising PGAP2 Novel Exon and Exon 4. (**c**) PGAP2 Novel Exon and Exon 4 qPCR expression in 22Rv1 vehicle and R1881 treated cells.

We next asked whether we can detect the novel exon to exon 4 junction in LN-95 and 22Rv1 cells, which both endogenously express small amounts of AR-V7. In LN-95 cells, we depleted AR-V7 using siRNA and measured expression by qPCR. Figure 6a shows the depletion of AR-V7 after treating LN-95 cells with V7 siRNA. Depletion of V7 eliminated expression of the novel exon linked to exon 4 (Fig. 6b), and treatment with R1881 induced expression many fold relative to basal (AR-V7 dependent) expression. 22Rv1 cells express multiple splice variants and AR-V7 is only a fraction of this pool ^9^, so depletion of AR-V7 alone would not be very informative. However, Fig. 6c shows a huge induction in novel exon to exon 4 junction expression in R1881 treated 22Rv1 cells with respect to vehicle treated cells supporting the concept that this novel exon generally is induced by AR isoforms.

### Alternative first exon event in TPD52

**Figure 7:**
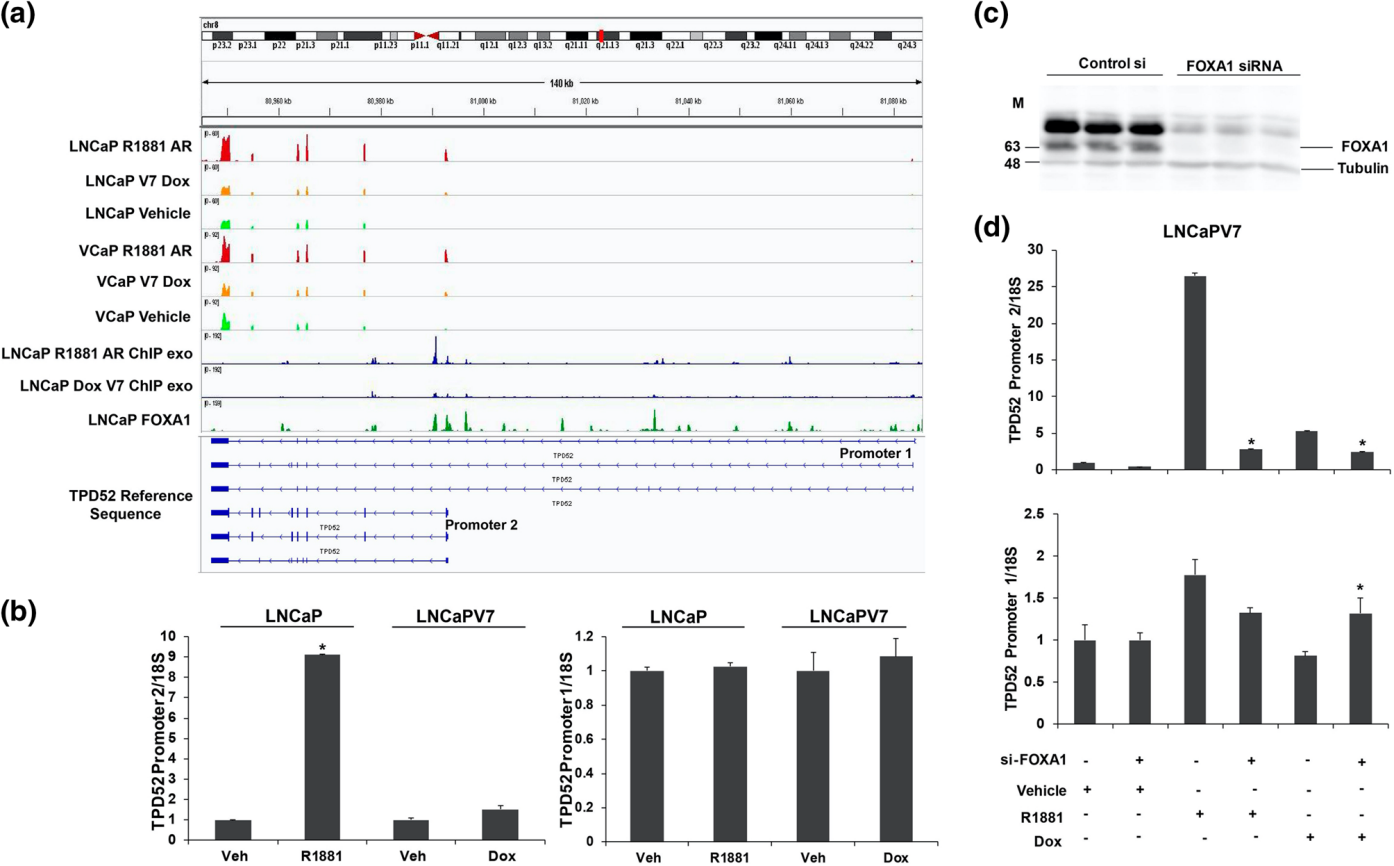
Alternative first exon event in TPD52. (**a**) IGV RNA expression reads of both isoforms of TPD52 in LNCaP Vehicle, LNCaP R1881, LNCaP-AR-V7 Dox treated samples, VCaP Vehicle, VCaP R1881, and VCaP-AR-V7 Dox treated samples, ChIP-exo tracks in LNCaP R1881 and LNCaP-AR-V7 Dox treated samples and a FOXA1 ChIP track in the LNCaP cell line. (**b**) To analyze the expression of the promoter 2 and the promoter 1 isoforms, LNCaP and LNCaP-AR-V7 cells were transferred to CSS medium and treated with vehicle, R1881, or Dox for 24 h and RNA was isolated and subjected to qPCR. (**c**) LNCaP-AR-V7 cells were treated with control si or FOXA1-siRNA and cells were harvested at 72 h for western blotting and probed with FOXA1 and tubulin antibodies. (**d**) LNCaP-AR-V7 cells were treated with control si or si-FOXA1 for 48 h followed by treatment with vehicle, R1881, or Dox for an additional 24 h. RNA was isolated subjected to qPCR with primers for promoter 2 and promoter 1 isoforms.

The PrLZ form of Tumour Protein D52 (TPD52) has a well-characterized role in prostate cancer progression^37–39^. Androgen driven selection of an alternative promoter in the TPD52 gene switches from promoter 1 (extended) to alternative promoter 2 (short) and this alternative promoter 2 is known to produce a protein isoform known as PrLZ^31^. Because AR is known to preferentially induce transcription from an alternative first exon^31^, we sought to determine whether AR-V7 also induced this form. Our RNA-seq data also suggested that TPD52 was induced by R1881 and that there also was preferential usage of the internal (promoter 2) rather than the extended 5′ promoter (promoter 1) resulting in different first exons. Figure 7a shows the IGV expression reads for TPD52 for both cell models. Whereas there are increased reads from the internal promoter and downstream exons in response to R1881, AR-V7 does not appear to alter expression. Measurement of the promoter 2 isoform by qPCR in the LNCaP model showed preferential induction by R1881 with no change in expression of the promoter 1 form (Fig. 7b), whereas AR-V7 had little effect on the expression of either form. Our previous studies suggested that preferential regulation of target genes by AR can require FOXA1^33^. An analysis of previously published FOXA1 binding to this gene using IGV showed multiple FOXA1 binding sites^40^ (Fig. 7a) surrounding the alternate exon and analysis of AR isoform binding to this gene using our ChIP-exo data showed more AR binding at one of these sites consistent with increased expression, but no notable AR-V7 binding in the region near the alternate exon (Fig. 7a). To test the role of FOXA1, LNCaP-AR-V7 cells were treated with control or FOXA1 siRNA followed by treatment with R1881 or Dox and cells harvested for western blotting and qPCR. The FOXA1 siRNA substantially reduced the expression of FOXA1 (Fig. 7c). It dramatically reduced the R1881 mediated induction of the promoter 2 form, with little effect on the promoter 1 form (Fig. 7d). There was a slight reduction in promoter 2 form in Dox treated cells, but there also was a reduction in basal expression of this form in response to the depletion of FOXA1 (Fig. 7d). Surprisingly, there was a small but consistent increase across multiple experiments in the promoter 1 form in cells depleted of FOXA1 and treated with Dox.

## Discussion

The best-characterized function of AR is its capacity to regulate transcription through binding to DNA. The diversity of gene products also is regulated through various forms of alternative splicing and differential exon usage. AR also plays a role in differential exon usage and alternative splicing^26,31^. The constitutively active AR splice variants that appear in CRPC also regulate transcription and there is evidence that although many of the actions of AR and its variants are similar, each can also regulate unique targets. Thus, we sought to determine whether their capacity to regulate splicing also differs. Our RNA-seq data derived from LNCaP and VCaP cell line models showed that not only do AR and AR-V7 differentially regulate expression of a subset of target genes but that both induce alternative splicing of mRNAs (Fig. 2a–c). Moreover, there are substantial differences in the splicing events induced by the two isoforms. In the VCaP model, approximately 26% of the AR-V7 regulated events at ψ greater than 0.2 are also regulated by AR and the overlap increases to 35% when no limit on ψ is imposed (Fig. 2d–e). The overlap for the LNCaP model is somewhat lower with 25% of AR-V7 events found to be regulated by R1881 when ψ level is not considered. Thus, the overlap between the two isoforms within a cell line is greater than isoform specific overlap between the cell lines (discussed earlier). This perhaps is not surprising because others have failed to find a high degree of overlap between overall mediated AR-V7 mediated transcription in different cell lines. For example, Chen et al. found a 11% overlap of AR-V7 upregulated genes (33/292) in 22Rv1 cells compared to LN-95 and a 17% overlap (33/192) when comparing LN-95 with 22Rv1^41^.

The PGAP2 exon skipping/inclusion event (exon 4) was chosen because it occurred in both cell line models and the Sashimi plot (Fig. 3a) suggested that AR-V7 preferentially induced inclusion of exon 4. Although qPCR of the exon 4–5 junction confirmed an induction by both isoforms, there was only a modest preference for AR-V7. Moreover, when a gel-based assay was used to probe for fragments that span exons 3–5, there was very little product relative to the skipped form, and no AR-dependent induction was found (Fig. 3c). When we analysed the RNA-seq tracks, we found that there was a novel transcribed region between exons 3 and 4, and this region was preferentially increased by AR-V7 (Fig. 4a). Analysis by qPCR confirmed that the region encompassing this novel exon and exon 4 was highly induced by AR-V7 and to a lesser extent by AR. An examination of reads containing a portion of this novel sequence as well as portions of other exons showed that the novel exon was primarily linked to exon 4 and to a lesser extent to further downstream exons including exon 5 (Fig. 4a). The absence of upstream reads linked to the novel exon suggested that this is a new transcription start site consistent with our failure to see an induced band of appropriate size when primers in exons 3 and 5 were used to detect RNA (Fig. 3c). Our ChIP-exo data showed that both AR isoforms bind in the region of the novel exon (Fig. 5a) and this was confirmed by ChIP-qPCR (Fig. 5b). However, the extent of binding is not sufficiently different to explain the differential induction of the novel exon by AR-V7. There is an upstream binding site unique to AR (Fig. 5a) that may alter induction at the novel exon. Alternatively, AR-V7 may recruit some unique factor(s). The novel exon is 75 bp long and is in-frame with the downstream coding sequence. It does not encode a methionine in any reading frame, so initiation of translation should begin in the downstream exon. The functional significance of a truncated protein is unknown. However, mutations resulting in Mabry syndrome are in a region common to all isoforms^42^ suggesting that this truncated form may be functional. We sought to test the protein expression levels of PGAP2 by western blotting with three different antibodies but were unable to detect the endogenous levels of full-length PGAP2 or the isoform containing novel exon in LNCaP AR-V7 cells.

We also studied alternative promoter usage in the TPD52 gene since previous reports had shown that AR preferentially induced transcription from promoter 2, downstream of promoter 1. The protein product produced from promoter 2, has been associated with worse outcomes in prostate cancer^39,43^. Moreover, it is expressed at higher levels in prostate cancer and even in prostatic epithelial neoplasia compared to normal or benign prostate epithelia^44^. We confirmed the preferential induction from promoter 2 both by examining our RNA Seq tracks and by qPCR of products initiated at promoter 1 or promoter 2. Interestingly, AR-V7 did not induce transcription from promoter 2. The same transcriptional pattern was observed in the VCaP model. Whereas our LNCaP model ChIP-exo data showed that AR exhibited binding to a region within the first intron following promoter 2, AR-V7 did not (Fig. 7a). Some of our previous studies of differential transcription showed that in some cases, FOXA1 facilitated AR-dependent transcription^33^. Analysis of FOXA1 binding in the region using a previously published FOXA1 binding track showed that there is a FOXA1 binding site at the same location as the AR site (Fig. 7a). The depletion of FOXA1 abrogated the AR-dependent induction from promoter 2 with little effect on transcription from promoter 1 (Fig. 7d) suggesting that the physical/functional interaction of AR with FOXA1 is critical for this differential activity.

In conclusion, we have found AR isoform-specific effects on the regulation of specific variants of gene transcripts. It is the combination of the level of expression and isoform that determines the biological outcome. Whereas the overall induction of PGAP2 by AR-V7 as assessed by RNA-seq is 2.66-fold, induction of the isoform containing the novel exon is more than 50-fold. In the case of TPD52, induction by R1881 measured by RNA-seq is 3.3-fold, but induction from promoter 2 is tenfold (Fig. 7b). Although the functional implication of the alternative mRNA isoforms identified in this study is largely unexplored, the results highlight the importance of analysis of both overall AR isoform mediated gene regulation and their actions at the mRNA isoform level. Further studies are needed to correlate the expression of specific splice variants with disease phenotypes and clinical outcomes.

## Materials and methods

### Cell lines and chemicals

LNCaP, VCaP and 22Rv1 cell lines were obtained from the American Type Culture Collection (ATCC) and cultured according to ATCC recommendations. LN-95 cells were obtained from Dr. Jun Luo, Johns Hopkins University, and cultured in phenol red-free RPMI 1640 medium with 10% Charcoal Stripped Serum (CSS). The LNCaP-AR-V7-pHage and VCaP-AR-V7-pHage cell lines^33^ were grown under the same conditions as the parental lines, but G418 was included to prevent the loss of the lentivirus. All cell lines were authenticated by STR profiling and maintained in culture for up to 3 months before a new batch was thawed. Cells were routinely tested for mycoplasma. Cell culture medium was purchased from Gibco, a division of Thermo Fisher Scientific (Waltham MA). Fetal Bovine Serum (FBS) and Charcoal Stripped Serum (CSS) were purchased from Sigma Aldrich, St. Louis, MO. CSS was also prepared in the cell culture core. R1881 and Doxycycline (Dox) were purchased from Sigma Aldrich. Unless specified, all other chemicals are reagent grade.

### Western blot analysis

LNCaP-AR-V7-pHage, VCaP-AR-V7 pHage and LN-95 cells were lysed by three cycles of freeze/thaw treatment using a lysis buffer containing 0.01 M Tris, 1 mM EDTA, 12 mM monothioglycerol pH 7.6, 0.4 M NaCl and protease inhibitors (Roche) and the supernatant was collected from the lysate after centrifugation at 12,000 rpm for 5 min. The concentration of proteins was determined by the Bradford protein assay reagent (GenDEPOT). A 7.5% SDS gel was used to resolve 20 μg of proteins. Proteins were then transferred to a Protran nitrocellulose membrane (Perkin Elmer, Waltham, MA) and blots were incubated using the previously described anti-AR antibody AR441 (RRID AB_11000751) at a 1:1000 dilution^32^, β-tubulin antibody (RRID AB_309885) at a 1:10,000 dilution, or a FOXA1 antibody (RRID: AB_2104842) at a 1:1000 dilution. Proteins were detected using horseradish peroxidase-conjugated secondary anti-mouse (GenDEPOT) antibody at 1:10,000 and ECL2 western blot substrate (Pierce, ThermoScientific, IL).

### Transient RNA interference

Knock-down of AR-V7 and FOXA1 expression was performed using the transient transfection of siRNA against the gene in comparison to non-targeted negative control siRNA. Synthesized oligo duplexes were purchased from Sigma-Genosys, The Woodlands, TX, and the sequences are listed in STable 2. Cells were transferred to medium containing CSS and transfected with 40 nM siRNA using the Lipofectamine RNAiMAX reagent (Invitrogen, Rockville IL) according to the manufacturer’s instructions for 72 h. Hormone and Dox treatments were added for the final 24 h and samples were then harvested using PureXtract RNAzol (GenDepot, Barker, TX) solution for RNA extraction. All experiments have been performed a minimum of 3 times with biological triplicates in each experiment. A representative experiment is shown.

### Semi-quantitative PCR

PureXtract RNAzol solution (GenDEPOT, Barker, TX) was used to prepare RNA from the treated LNCaP, LNCaP-AR-V7, VCaP, and VCaP-AR-V7 cells. cDNA Synthesis Master Mix (GenDEPOT) was used to reverse transcribe the total RNA (0.5 μg) to make cDNA to measure target genes. AmfiSure Taq DNA Polymerase (GenDEPOT) was used for PCR amplification. Primers used for amplification are listed in the primer STable 1. 1X Tris–borate–EDTA (TBE) buffer was used for gel electrophoresis. A 1% agarose gel was prepared with 1X TBE buffer and 0.5 μg/mL ethidium bromide. The 1 Kb Plus DNA ladder (Invitrogen) was used for estimating molecular weights. Gels were visualized on the Carestream Molecular Imaging gel documentation system.

### Quantitative RT-PCR

PureXtract RNAzol solution (GenDEPOT, Barker, TX) was used to prepare RNA from the treated LNCaP, LNCaP-AR-V7, VCaP, VCaP-AR-V7, 22Rv1 and LN-95 cells. cDNA Synthesis Master Mix (GenDEPOT) was used to reverse transcribe the total RNA (0.5 μg) to make cDNA to measure target genes. Target gene expression was analysed using the primers (STable 1) and SYBR Green PCR Master Mix (Applied Biosystems by Life Technologies, TX) with the Step One Plus Real-Time PCR System (Applied Biosystems, Foster City, CA). qPCR data were analysed by the relative standard curve method, using 18S rRNA as the reference transcript and treatment values were normalized to the average expression of the vehicle-treated cells. All experiments have been performed a minimum of 3 times with biological triplicates in each experiment. A representative experiment is shown.

### Chromatin immunoprecipitation

ChIP assays were performed as previously described^33^ using AR antibody (RRID: AB_2793341) or rabbit IgG antibody (Invitrogen). Immunoprecipitated DNA fragments were measured by qPCR using the primer sequences listed in the Supplementary file.

### RNA-seq alignment and identification of alternatively spliced genes

For each sample, reads were quality trimmed using Trim Galore! and then aligned to the hg19 genome using HISAT2^45,46^. Gene expression was quantified using feature counts against the GENCODE gene reference. Differentially expressed genes were detected using edgeR, with significance achieved at FDR-adjusted p-value < 0.05 and fold change exceeding 1.25x. The Mixture-of-Isoforms (MISO) model was applied to identify alternatively spliced genes and differential used exons following the developer's guidelines^47^. We considered alternative splicing effects for a Bayesian Factor (BF) ≥ 3 and a percent spliced in (psi (ψ)) difference of at least 0.2.

### Analysis of public prostate cancer datasets

RNAseq and ChIP-exo track expression datasets used are listed in STable 3.

### Statistical analysis

All experiments have been performed a minimum of three times and a representative experiment was shown. The Student’s *t*-test was used to calculate statistical significance (*P* < 0.05) using PASW SPSS 18 software**.**

## Supplementary Information

Supplementary Information.
