# Author Correction: Androgen receptor and its splice variant, AR-V7, differentially induce mRNA splicing in prostate cancer cells

**DOI:** 10.1038/s41598-021-90079-9

**Published:** 2021-05-11

**Authors:** Manjul Rana, Jianrong Dong, Matthew J. Robertson, Paul Basil, Cristian Coarfa, Nancy L. Weigel

**Affiliations:** 1 Department of Molecular and Cellular Biology, Baylor College of Medicine, Houston, TX 77030 USA; 2 Dan L. Duncan Cancer Center, Baylor College of Medicine, Houston, TX 77030 USA; 3 Present Address: Mercury Data Science, Houston, TX 77098 USA

Correction to: *Scientific Reports* 10.1038/s41598-021-81164-0, published online 14 January 2021

Paul Basil was omitted from the author list in the original version of this Article. This has now been corrected in the PDF and HTML versions of the Article, and in the accompanying Supplementary Information file.

The Author Contributions section now reads:

NW, MR, and CC conceived the study. MR performed the experiments and PB prepared and provided ChIP and siRNA samples for qPCR analyses. JD, MJR, MR, PB, and CC performed data analysis and prepared figures. MR, CC, and NW wrote the manuscript. All authors reviewed the manuscript.

The Acknowledgements section now reads:

We thank Dr. Thomas Cooper for his valuable suggestions. We thank W.E. Bingman III for his technical assistance. We also thank the Molecular and Cellular Biology tissue culture core for assistance in the growth and maintenance of cell cultures and the BCM protein and monoclonal antibody production core, which is supported by the P30 Cancer Center support grant (NCI-CA125123). This work was supported by DAMD W81XWH-17-1-0236 and CPRIT RP150648. This project also was partially supported by The Cancer Prevention Institute of Texas (CPRIT) RP170005, NIH P30 shared resource grant CA125123, and NIEHS grant 1P30ES030285.

