# Supplementary material for: Androgen receptor and its splice variant, AR-V7, differentially induce mRNA splicing in prostate cancer cells
Source: Sci Rep. 2021 Jan 14;11:1393. doi: 10.1038/s41598-021-81164-0 (PMC7809134; doi:10.1038/s41598-021-81164-0)
Supplement: Supplementary file 1 — Supplementary Information. [file 41598_2021_81164_MOESM1_ESM.pdf]

Androgen Receptor and its Splice Variant, AR-V7, Differentially Induce mRNA Splicing in Prostate Cancer Cells

Manjul Rana[1], Jianrong Dong[1,2,3], Matthew J Robertson[1,2], Paul Basil[1], Cristian Coarfa[1,2*], Nancy L Weigel[1*]

[1]Department of Molecular & Cellular Biology, [2]Dan L. Duncan Cancer Center, Baylor College of Medicine, Houston, TX 77030, USA, [3] Present address: Mercury Data Science, Houston, TX 77098, USA

[*] Correspondence: Nancy L. Weigel

Email: nweigel@bcm.edu

Tel: +1 713 798-6234

Fax: +1 713 790-1275

List of Figures and tables in supplementary data

STable 1- List of Primers

STable 2- List of siRNA sequences

STable 3- List of GEO datasets used

SFig. 1- Description of PGAP2 isoform containing novel exon: The novel exon is present between exons 3 and 4 in LNCaP cell line model (Fig. 4a and SFig. 1a). This novel exon is of 75 bp long (SFig. 1a, shown in blue) and it is in frame with the following exons (SFig. 1b). Exon 4 contains a methionine in different reading frames and the novel exon does not contain a methionine in any reading frame, so initiation of translation would presumably begin in a known exon (exon 4) (SFig. 1b).

Full-length blots/gel pictures

Figure a. Full-length western blot for main Figure 1b in the manuscript.

Figure b. Full-length western blot for main Figure 1e in the manuscript.

Figure c. Full-length western blot for main Figure 6a in the manuscript.

Figure d. Full-length western blot for main Figure 7c in the manuscript.

STable 1: List of Primers

Gene	Sequence
**qPCR primers**	
PSA FP	5' ACCTGCACCCGGAGAGCT 3'
PSA RP	5' TCACGGACAGGGTGAGGAAG 3'
EDN2 FP	5' CTGCAGACGTGTTCCAGAC 3'
EDN2 RP	5' AGGCTCTTGACTGTGGAAATG 3'
18S FP	5' ACCGCAGCTAGGAATAATGGA 3'
18S RP	5' GCCTCAGTTCCGAAAACCA 3'
PGAP2 Exon4 FP	5' CCTGTGTTCGGCTTCTTCTT 3'
PGAP2 Exon5 RP	5' CACCGAGGGCAGGTAATTG 3'
PGAP2 Novel Exon and Exon4 FP	5' AGTCTGGCTACAACCTTCAAC 3'
PGAP2 Novel Exon and Exon4 RP	5' AAGCGAAGCCGGAACAA 3'
PGAP2 ChIP FP	5' CCTCAGCCACTATCACTTCTTC 3’
PGAP2 ChIP RP	5’ GCCCACAGTACCCAATTCT 3’
TPD52 Short FP	5' AGGACTACCAGTCCCCGTTT 3'
TPD52 Short RP	5' AGGGACTGGGTCTGTTCTCA 3'
TPD52 Extended FP	5' CGGGCTGGAGACATGGA 3'
TPD52 Extended RP	5' ATCGTGGCAGCAACATCTTC 3'
**Semi-quantitative PCR primers**	
PGAP2 Exon3 FP	5' TCTTCCACTTCAAGGAGACAAC 3'
PGAP2 Exon5 RP	5' GGAGACATAAGTGAGCACTAGC 3'
PGAP2 Novel Exon and Exon 5 FP	5' AGTCTGGCTACAACCTTCAAC 3'
PGAP2 Novel Exon and Exon 5 RP	5' GGAGACATAAGTGAGCACTAGC 3'

STable 2: List of siRNA sequences

siRNA	Sequence
siRNA FoxA1	GCACUGCAAUACUCGCCUU
siAR-V7	GGGUUGGCAAUUGCAAGCAUCUCAA

STable 3: List of GEO datasets used

Serial number	Description	Reference
	**ChIP-seq data**	
1	AR ChIP-exo	GSE143906
2	AR-V7 ChIP-exo	GSE143906
3	FOXA1 tracks in LNCaP	He, B. *et al.* 2014 (Ref. 40)
	**RNA-seq data**	
1	LNCaP AR-V7	GSE143905
2	LNCaP AR R1881	GSE143905
3	LNCaP Vehicle	GSE143905
4	VCaP AR-V7	GSE143905
5	VCaP AR R1881	GSE143905
6	VCaP Vehicle	GSE143905

SFig.1

a)

..........GCC ACA CAC TGT GGG AGT CTT AGA ATA GTC CCA GTC TGG CTA CAA CCT TCA ACC CAA GAC

.... A T H C G S L R I V P V W L Q P S T Q D

CCG GAT TCT TCA GCA CCG AGT GTG AGT GTT GCC ACG CCC TGC AGG ATG TTC TCT GCG GCC ....

P D S S A P S V S V A T P C R Met F S A A ....

b)

AGT CTT AGA ATA GTC CCA GTC TGG CTA CAA CCT TCA ACC CAA GAC CCG GAT TCT

TCA GCA CCG AGT GTG AGT GTT GCC ACG CCC TGC AGG ATG TTC TCT GCG GCC ....

Met F S A A ....

SFig.1. Description of PGAP2 isoform containing novel exon. **a)** Novel exon (blue) is present between exon 3 (red) and exon 4 (green). Shown are the nucleotide and possible amino acid sequences. **b)** Predicted Translation Sequence of the PGAP2 Isoform containing novel exon. Novel exon nucleotide sequence is shown in blue and nucleotide sequence of Exon 4 is shown in green. The novel exon does not contain a methionine in any reading frame, so initiation of translation would presumably begin in a known exon.

Figure a

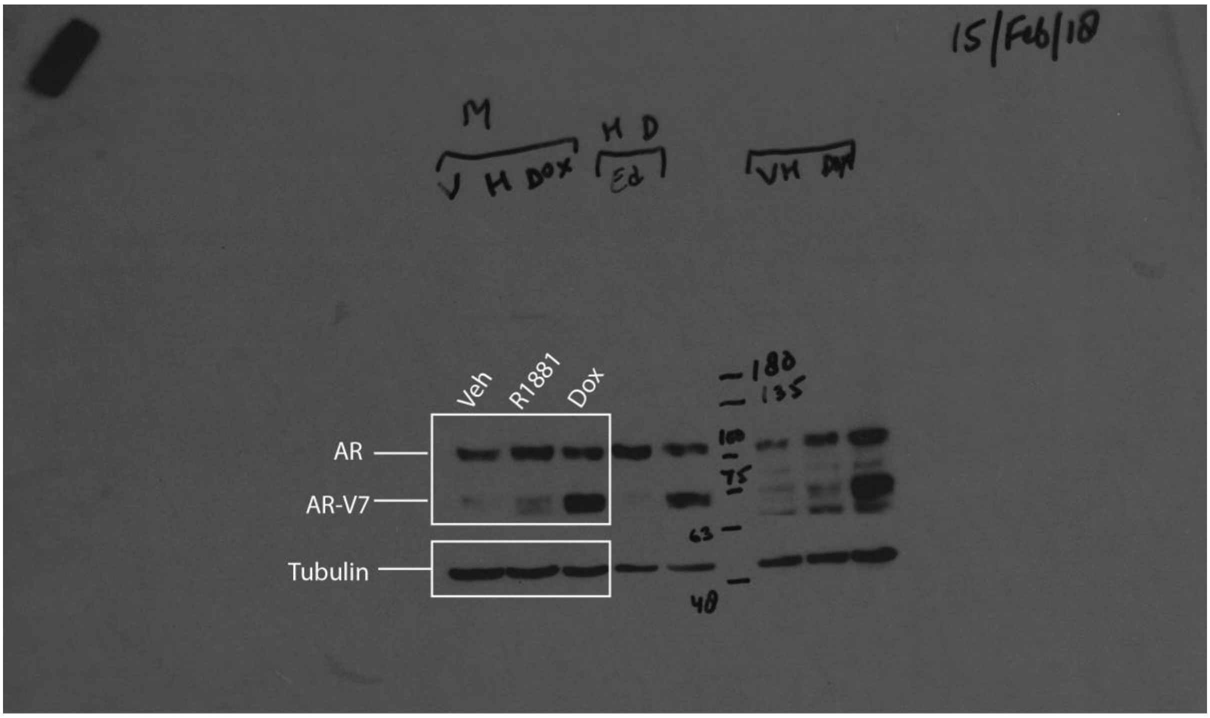

Figure a. Full-length western blot for main Figure 1b in the manuscript.

Figure b

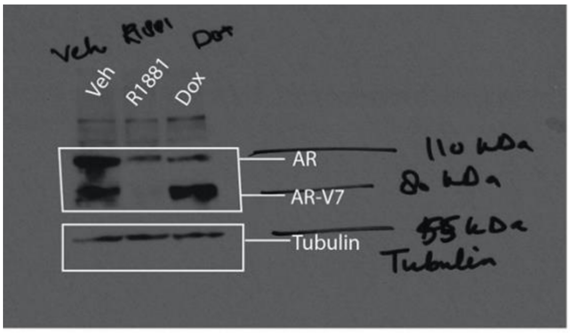

High exposure

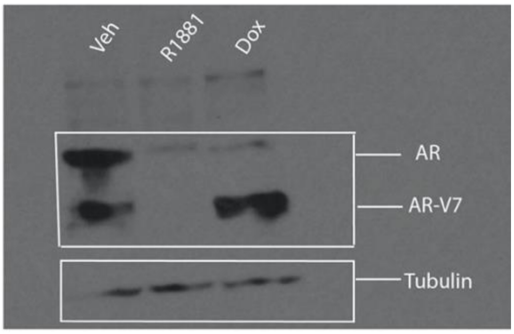

Low exposure

Figure b. Full-length western blot for main Figure 1e in the manuscript.

Figure c

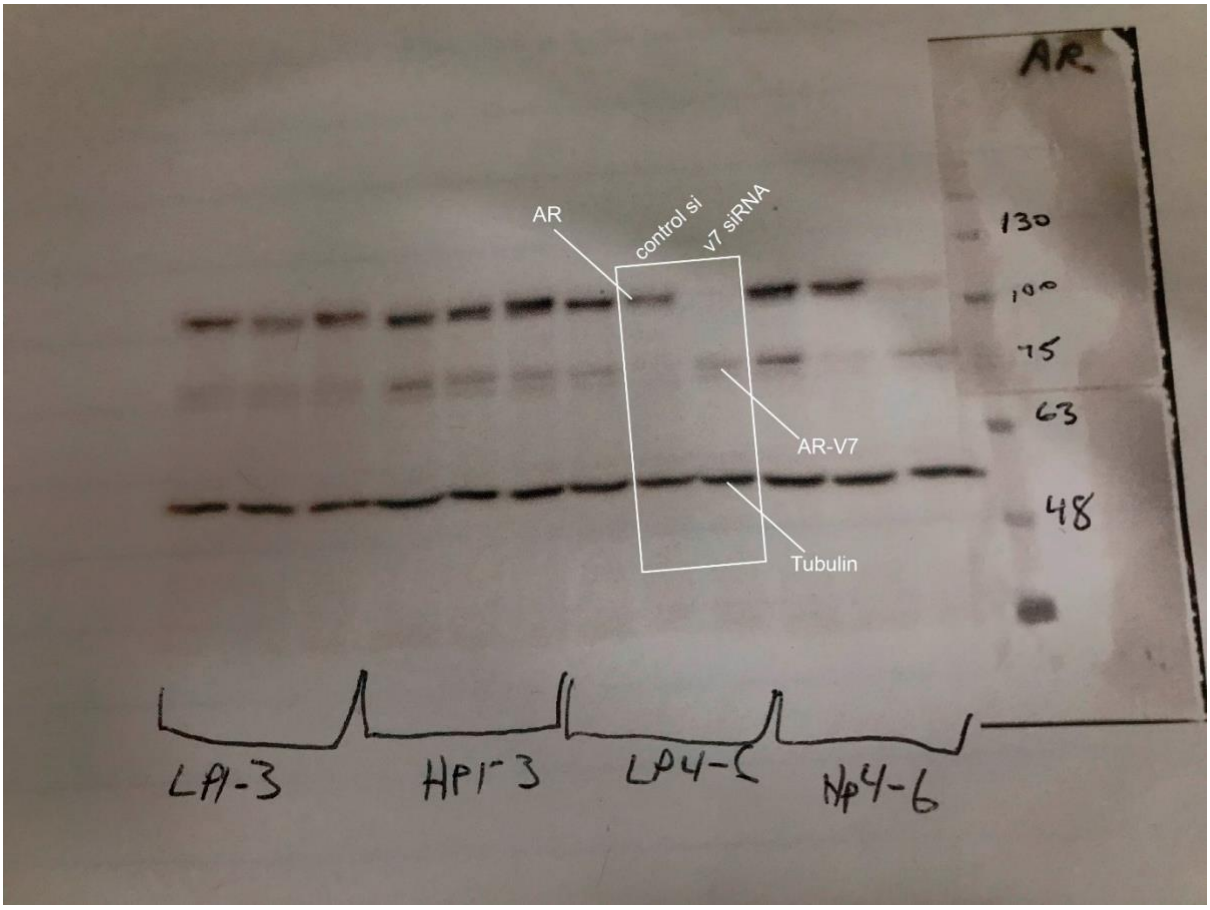

Figure c. Full-length western blot for main Figure 6a in the manuscript.

Figure d

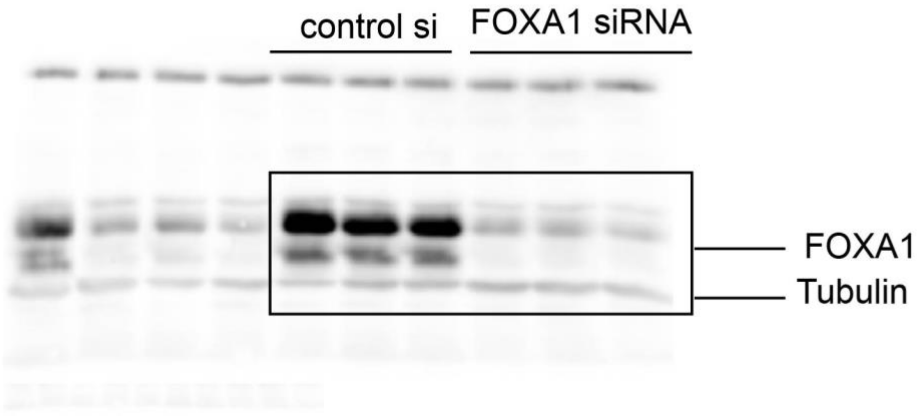

Figure d. Full-length western blot for main Figure 7c in the manuscript.
